# Fc-Based Recombinant Henipavirus Vaccines Elicit Broad Neutralizing Antibody Responses in Mice

**DOI:** 10.3390/v12040480

**Published:** 2020-04-23

**Authors:** Yaohui Li, Ruihua Li, Meirong Wang, Yujiao Liu, Ying Yin, Xiaodong Zai, Xiaohong Song, Yi Chen, Junjie Xu, Wei Chen

**Affiliations:** Laboratory of Vaccine and Antibody Engineering, Beijing Institute of Biotechnology, Beijing 100071, China; 18612736076@163.com (Y.L.); ilovebones@163.com (R.L.); lullabywangmeirong@outlook.com (M.W.); yjliu418@outlook.com (Y.L.); yinying1028rr@hotmail.com (Y.Y.); zaixiaodong@163.com (X.Z.); m13810676152@163.com (X.S.); chenyi510@icloud.com (Y.C.)

**Keywords:** henipavirus, vaccine, attachment glycoprotein

## Abstract

The genus *Henipavirus* (HNVs) includes two fatal viruses, namely Nipah virus (NiV) and Hendra virus (HeV). Since 1994, NiV and HeV have been endemic to the Asia–Pacific region and responsible for more than 600 cases of infections. Two emerging HNVs, Ghana virus (GhV) and Mojiang virus (MojV), are speculated to be associated with unrecognized human diseases in Africa and China, respectively. Despite many efforts to develop vaccines against henipaviral diseases, there is presently no licensed human vaccine. As HNVs are highly pathogenic and diverse, it is necessary to develop universal vaccines to prevent future outbreaks. The attachment enveloped glycoprotein (G protein) of HNVs mediates HNV attachment to the host cell’s surface receptors. G proteins have been used as a protective antigen in many vaccine candidates for HNVs. We performed quantitative studies on the antibody responses elicited by the G proteins of NiV, HeV, GhV, and MojV. We found that the G proteins of NiV and HeV elicited only a limited cross-reactive antibody response. Further, there was no cross-protection between MojV, GhV, and highly pathogenic HNVs. We then constructed a bivalent vaccine where the G proteins of NiV and HeV were fused with the human IgG1 Fc domain. The immunogenicity of the bivalent vaccine was compared with that of monovalent vaccines. Our results revealed that the Fc-based bivalent vaccine elicited a potent antibody response against both NiV and HeV. We also constructed a tetravalent Fc heterodimer fusion protein that contains the G protein domains of four HNVs. Immunization with the tetravalent vaccine elicited broad antibody responses against NiV, HeV, GhV, and MojV in mice, indicating compatibility among the four antigens in the Fc-fusion protein. These data suggest that our novel bivalent and tetravalent Fc-fusion proteins may be efficient candidates to prevent HNV infection.

## 1. Introduction

*Henipavirus* (HNV) is a genus of paramyxovirus and comprises five well-established species [1]. Nipah virus (NiV) and Hendra virus (HeV) are highly pathogenic and can cause fatal human diseases. The *Pteropus* bat species appear to be the major natural reservoir hosts for henipaviruses (HNVs), and all bat isolates of HeV and NiV have been derived from the genus *Pteropus*’s bats [2,3,4,5,6]. HeV was first isolated after an outbreak in the town of Hendra, Australia in 1994. An NiV outbreak was first reported in 1998 in Malaysia and Singapore, but the virus was first isolated in 1999 [7]. During the NiV outbreaks in Malaysia, pigs were the intermediate as well as amplifying hosts for the virus, and research showed a direct transmission of Nipah virus from pigs to human beings [8]. Since then, almost every year, outbreaks of HeV and NiV have caused severe infections in humans. The latest NiV outbreak occurred in Kerala, India, in May 2018, when 18 people were infected, and the case fatality rate was 91% [9]. Two genetically distinct strains have been described, these two strains are named Malaysia strain (NiV-My) and Bangladesh strain (NiV-Bd). The nucleotide similarity between the NiV-My and NiV-Bd strains is 91.8%, with similarities between proteins at ≥92% [10]. NiV-Bd is more pathogenic in African green monkeys under identical experimental conditions [11]. Cedar virus (CedV) was isolated in Australian bats and found to be non-pathogenic to ferrets and guinea pigs [12]. Two new HNVs have been discovered recently and have a concerning potential to cause human disease. Ghana virus (GhV) was found in bats in Africa [13,14], whereas Mojiang virus (MojV) was found in rats in an abandoned mine in Yunnan Province, China, and is thought to be associated with an unknown disease that killed four miners [15]. GhV and MojV have been fully sequenced but have not yet been isolated [13,15].

Nipah and henipaviral diseases are now in the WHO R&D blueprint list of epidemic threats requiring urgent R&D action [16]. There is currently no approved human vaccine for HeV and NiV. HNVs infect their host cells via attachment (G) and fusion (F) viral envelope glycoproteins. The G protein mediates the attachment to the host cell’s surface receptors, and the F protein mediates the fusion of the virus–cell membranes. The G proteins of HNVs are considered a major immunodominant target for antibody responses in animals and humans, which suggests that the G proteins of HNVs are ideal vaccine immunogens. Various vaccine platforms have been applied to HNV vaccine design [17,18], such as the replication-deficient simian adenovirus vector ChAdOX1 and the vesicular stomatitis virus (VSV) [19,20]. Recombinant NiV vaccines based on the canarypox vaccine vector ALVAC have been shown to protect pigs from the NiV challenge [21]. A new vaccine platform based on the recombinant bovine herpesvirus-4 vectors expressing G or F has been recently shown to induce potent antibody and T cell responses in pigs [22]. The most extensively studied NiV vaccine is a subunit vaccine based on a soluble HeV G protein (HeV-sG). HeV-sG is capable of eliciting potent cross-reactive neutralizing antibody responses against NiV and HeV in cats, ferrets, and monkeys [23,24,25,26]. An HeV-sG vaccine (Equivac^®^ HeV, Zoetis, Parsippany-Troy Hills, New Jersey, USA) was licensed for use in horses in Australia. However, the same vaccine failed to protect pigs against both HeV and NiV [27]. In addition, vaccination with a simian adenovirus-based vaccine encoding the NiV-G protein protected Syrian hamsters against the lethal challenge with NiV but not HeV [19]. For GhV and MojV, studies have shown that the antigenicity of their G proteins is distinct from that of NiV/HeV [13,28].

EphrinB2 has been proven to be a functional receptor of NiV, HeV, and GhV [13,29], and ephrinB3 can also be recognized by the G protein of NiV and HeV [30]. The host range of HNVs is broad and is facilitated by their use of conserved ephrin-B2 or/and B3 as the cellular receptors [31,32], increasing the possibility of further potential large-scale outbreaks. Therefore, it may be useful to further study the cross-reactive antibody response elicited by the HNV-G proteins and combine antigens with distinct immunogenicity to develop a universal vaccine against HNVs.

Fc-based fusion proteins consist of an immunoglobin Fc domain that is linked to another protein or peptide. The fused partner can be any proteinaceous molecule of interest, such as antigens of pathogens. The Fc domain folds independently and can improve the solubility and stability of the partner molecule both in vitro and in vivo [33]. The Fc domain can bind to the fc receptors on antigen-presenting cells and promote antigen delivery and the Fc region allows for cost-effective purification by protein-G/A affinity chromatography during manufacture [33,34]. In the research on HIV, respiratory syncytial virus, Epstein–Barr virus, and Ebola virus, fusion with Fc proved to improve the immune response stimulated by the recombinant proteins [35,36,37,38]. The use of Fc-fusion protein-based drugs can also prove the safety of this technology [33].

In the present study, the amino acid sequence homology and phylogeny of the G proteins from HNVs were analyzed, and the cross-reactive antibody responses triggered by the G proteins were comprehensively investigated. We designed and expressed a bivalent Fc-fusion protein that can stimulate the neutralizing antibody response against HeV and NiV. Compared with the monovalent soluble G protein, the bivalent Fc-fusion protein can produce a broad antibody response. We also developed a heterodimeric Fc-fusion protein using the “knobs-into-holes” technology [39]. This heterodimeric protein contains four antigens and stimulates potent antibody responses against the G proteins of NiV, HeV, GhV, and MojV. These results demonstrate that the novel bivalent and tetravalent vaccines are promising broad-spectrum henipaviral disease vaccine candidates.

## 2. Materials and Methods

### 2.1. Phylogenetic Analysis of the G Proteins

The amino acid sequences of 54 HNV-G proteins were obtained from the Genebank database. All sequences were aligned using the MEGA 7 software. The evolutionary history was inferred using the maximum likelihood method based on a JTT matrix-based model [40]. The bootstrap consensus tree inferred from 1000 replicates [41] was taken to represent the evolutionary history of the analyzed taxa. Branches corresponding to the partitions that reproduced in less than 50% of the bootstrap replicates were collapsed. The evolutionary analyses were conducted in MEGA 7 [42].

### 2.2. Protein Expression and Purification

Proteins were expressed using the Expi293™ expression system (Thermo Fisher Scientific, Waltham, MA, USA), which is a high-yield transient expression system based on suspension-adapted human embryonic kidney (HEK) cells. In brief, for the scalable transfection of the Expi293F™ cell lines in the Expi293™ expression medium (Gibco, Grand Island, NY, USA), 30 μg of plasmid was transfected into 30mL Expi293F cells at a cell density of approximately 4.5–5.5 × 10^6^ viable cells/mL. The cells were incubated at 37 °C with a humidified atmosphere of 8% CO_2_ on an orbital shaker. After 3 days, the cell supernatant was collected. The supernatant was centrifuged at 3000g for 10 min and filtered through a 0.45 μm filter (Thermo Fisher Scientific). All purified proteins were concentrated using a 30kDa ultrafiltration tube (Millipore, Bedford, MA, USA), and the buffer was replaced with PBS. The concentration of the protein was measured using a BCA kit (Pierce™ BCA Protein Assay kit, Thermo Fisher Scientific). Proteins were characterized using reduced and non-reduced SDS-PAGE [43].

#### 2.2.1. G Proteins of HNVs

The coding region for the HNV-G protein extracellular domains was codon-optimized and synthesized, and the tPA signal peptide and 6×his tag was added at the N-terminus. The genes were inserted into the pcDNA3.1 (+) mammalian expression vector (Invitrogen, Carlsbad, CA, USA). The proteins were purified using the HisTrap HP affinity chromatography column (GE Health Care, Chicago, IL, USA). G_NiV-My_ (Malaysia Nipah virus), G_NiV-Bd_ (Bangladesh Nipah virus), G_HeV_ (Hendra virus), G_GhV_ (Ghana virus), and G_MojV_ (Mojiang virus) were expressed and purified.

#### 2.2.2. Monoclonal Antibody M102.4

M102.4 is a monoclonal antibody that can neutralize NiV and HeV [44,45,46,47]. The variable region of M102.4 was combined with the human IgG1 constant region. The genes of the combined light chain and heavy chain were codon-optimized and synthesized. The genes were constructed into the pcDNA3.1 (+) vector. The plasmids of the light chain and heavy chain were co-transfected into Expi293 cells at a 1:1 ratio. M102.4 was purified using a HiTrap™ protein A HP affinity chromatography column (GE Health Care).

#### 2.2.3. Fc-Fusion Proteins

The bivalent Fc-fusion protein (Fc2HNV) contains the head domains of G_NiV_ and G_HeV_. The head domain of G_NiV_, the human IgG1-Fc domain, and the head domain of G_HeV_ were connected in sequence. These three domains were separated by two (Gly4Ser)3 flexible linkers. To obtain the secreted Fc-fusion protein, a tPA signal peptide was added at the N-terminus of the protein. The codon-optimized genes were synthesized and constructed into the pcDNA3.1 (+) vector. The Fc2HNV was expressed in the Expi293 suspension cells. Fc2HNV was purified using a HiTrap™ protein A HP affinity chromatography column (GE Health Care).

The bivalent Fc-fusion protein (Fc4HNV) contains the head domains of G_NiV_, G_HeV_, G_GhV_, and G_MojV_. To obtain the secreted Fc-fusion protein, a tPA signal peptide was added at the N-terminus of the protein. To construct the pcDNA3.1-Fc4HNV-chainA plasmid, the head domain of G_NiV_, the human IgG1-Fc domain, and the head domain of G_HeV_ were connected in sequence and separated by two (Gly4Ser)3 flexible linkers. To construct the pcDNA3.1-Fc4HNV-chainB plasmid, the head domain of G_GhV_, the human IgG1-Fc domain, and the head domain of G_MojV_ were connected in sequence and separated by two (Gly4Ser)3 flexible linkers. The Fc domains of the two chains carry “knobs-into-holes” mutations [39]. The pcDNA3.1-Fc4HNV-chainA and pcDNA3.1-Fc4HNV-chainB were co-transfected into Expi293 cells at a 1:1 ratio. In order to obtain the correct heterodimer, the two chains of Fc4HNV were inserted with a 6x His tag and a strep tag, respectively, and purified by Ni-affinity chromatography and strep-affinity chromatography in tandem.

### 2.3. Coupling of Purified G Glycoproteins to Microspheres

An amount of 50 μg of purified G protein was coupled to 1.25 × 10^7^ MagPlex microspheres (Luminex Corporation, Austin, TX, USA) using an xMAP Antibody Coupling Kit (Luminex Corporation). For the microsphere activation, 1.25 × 10^7^ of the stock microspheres was transferred to the recommended microcentrifuge tubes. The liquid was removed using a 1.5 mL tube magnetic separator (Luminex Corporation). The microspheres were washed once in dH_2_O. The washed microspheres were resuspended in 80 μL of 0.1 M sodium phosphate (monobasic, pH 6.2) by vortex and sonication for approximately 20 s. Subsequently, the microspheres were incubated in an activation buffer containing 5 mg/mL 1-ethyl-3-(3-dimethylaminopropyl) carbodiimide HCl (EDC) (Thermo Fisher Scientific) and 5 mg/mL *N*-hydroxysulfosuccinimide (*S*-NHS) (Luminex Corporation) for 20 min at 24 °C with shaking in the dark. The liquid was removed, and the purified G proteins were added. The microspheres and antigens were incubated for 2 h at 24 °C with shaking in the dark. The microspheres were washed twice with PBSA (PBS, 1% BSA) and resuspended in a 600 μL microsphere storage buffer (Luminex Corporation).

### 2.4. Multiplex Microsphere Receptor Binding Assay

The binding between the purified G proteins and ephrinB2 and ephrinB3 was measured to determine the ligand cross-reactivity. G protein-coupled magnetic microspheres were mixed after sonication such that all assays were multiplexed. A total of 1500 mixed microspheres were added per well. The recombinant mouse ephrin-B2 Fc chimera biotinylated protein (R&D systems, Minneapolis, MN, USA) and recombinant human ephrin-B3 Fc chimera biotinylated protein (R&D systems) were diluted in PBSA and added into the microsphere-containing wells with two replicates per concentration. The 96-well assay plate (Corning Inc, Corning, NY, USA) was incubated for 60 min at 24 °C on a plate shaker at 800 rpm. Streptavidin-R-phycoerythrin (SAPE) (Invitrogen) was diluted to a concentration of 12 µg/mL. A total of 10 µL of diluted SAPE was added to each well. The assay plate was incubated for 30 min at 24 °C on a plate shaker at 800 rpm. The supernatant was removed using a magnetic plate separator (Luminex Corporation). The plate was washed three times with PBSA, and the binding was measured using Luminex MAGPIX instrument (Luminex Corporation). The net median fluorescence intensities (MFI) were recorded and used to draw the binding curve.

### 2.5. Animal Immunization

Specific pathogen-free BALB/c mice (6–8 weeks, female) were immunized intramuscularly with 10 μg G_NiV-My_, G_NiV-Bd_, G_HeV_, G_GhV_, G_MojV_, Fc2HNV, or Fc4HNV at weeks 0 and 3. The adjuvants added were 200 μg of aluminium hydroxide adjuvant (InvivoGen, San Diego, CA, USA) and 20 μg of CpG ODN 1826 (InvivoGen). The volume of inoculum per mouse was 100 μL. The mice were sacrificed 42 days following the first immunization, and the serum was collected. All of the animal experiments in this study were approved by the Laboratory Animal Care and Use Committee of the Beijing Institute of Biotechnology (approval number: IACUC of AMMS-08-2018-001, date of approval is 10 July, 2018). Mice were sacrificed at the indicated time by CO_2_ inhalation. All efforts were made to minimize suffering.

### 2.6. Multiplex Microsphere Receptor Inhibition Assay

The inhibition of the binding to the ephrin receptors by the purified G proteins was measured to establish 50% inhibiting concentration (IC50) values for each serum sample. The G protein-coupled magnetic microspheres were mixed after sonication such that all assays were multiplexed. A total of 1500 microspheres was added to each well. The recombinant mouse ephrin-B2 Fc chimera biotinylated protein (R&D systems) or recombinant human ephrin-B3 Fc chimera biotinylated protein (R&D systems) were diluted in PBSA and added into the microsphere-containing wells at a final concentration of 25 ng/mL, and the serum was three-fold serially diluted from 1:20 with two replicates per dilution. The assay plate was incubated for 60 min at 24 °C on a plate shaker at 800 rpm. Streptavidin-R-phycoerythrin (Invitrogen) was diluted to a concentration of 12 μg/mL. Thereafter, 10 µL of diluted SAPE was added to each well. The assay plate was incubated for 30 min at 24 °C on a plate shaker at 800 rpm. The supernatant was removed using a magnetic plate separator (Luminex Corporation). The plate was washed three times with PBSA, and the binding was measured using a Luminex MAGPIX instrument (Luminex Corporation). The MFI was recorded for a four-parameter logistic curve fitting, and the IC50 was calculated based on this curve.

### 2.7. Enzyme-Linked Immunosorbent Assay (ELISA)

The cross-antibody response for G proteins from different viruses was investigated using BALB/c mice inoculated with G_NiV-My_, G_NiV-Bd_, G_HeV_, G_GhV_, and G_MojV_ proteins. All proteins were expressed in the Expi293 expression system and were purified using a HisTrap HP affinity chromatography column. Aliquots of 200 ng of G protein were added to the coating buffer (50 mM carbonate buffer, pH=9.6), put on a 96-well assay plate (Corning Inc), and incubated at 4 °C for 12 h. The supernatant was removed, and 100 μL of blocking buffer (PBS with 2% BSA) was added to each well. The plate was incubated at 37 °C for 1 h and washed 4 times with a wash buffer (PBS with 0.1% Tween20). The serum was three-fold serially diluted from 1:100 with two replicates per dilution and incubated for 1 h at 37 °C; then, it was washed 4 times with a wash buffer. The HRP-conjugated secondary goat anti-mouse IgG Fc or the HRP-conjugated secondary goat anti-human IgG H&L (Abcam, Cambridge, United Kingdom) were added at a concentration of 1:10000 and incubated for 1 h at 37 °C, plate was washed 4 times with a wash buffer. An aliquot of 100 µL of a TMB single-component substrate solution (Solarbio life sciences, Beijing, China) was added to each well. The plate was developed at 24 °C in the dark for 5 min before the addition of 50 µL of an ELISA stop solution (Solarbio life sciences) to each well. The absorbance was measured at 450 nm minus 630 nm. All values were recorded for the four-parameter logistic curve fitting, and the antibody titers were calculated based on this curve. The cut-off value was set to 2.1 times the value of the negative control.

### 2.8. Pseudotyped Virus Packaging

Codon-optimized, full-length G and F protein genes of NiV and HeV were inserted into the pcDNA3.1 vector to generate synthetic viral proteins. For the NiV pseudovirus (NiV-PP), the sequences of the G (Genebank protein ID: AAY43916.1) and F (AAY43915.1) proteins were used; for the HeV pseudovirus (HeV-PP), the sequences of the G (AEQ38071.1) and F (AEQ38070.1) proteins were used. By truncating the pcDNA3.1-NiV-F plasmid, 5 amino acids in the C-terminal intracellular region of the F protein were retained [48]; the pseudovirus made by the truncated F plasmid was named NiV-T5F-PP. By truncating the pcDNA3.1-HeV-F plasmid, 5 amino acids in the C-terminal intracellular region of the F protein were retained; the pseudovirus made by the truncated F plasmid was named HeV-T5F-PP. A total of 7.0 × 10^6^ 293T cells were inoculated in a 10 cm culture dish overnight at 37 °C with 5% CO_2_. The cells were maintained in a high-glucose DMEM (Gibco) supplemented with 10% FBS (Gibco), penicillin (100 IU/mL), and streptomycin (100 μg/mL). The pcDNA3.1-G and pcDNA3.1-F plasmids were co-transfected into 293T cells with the HIV backbone vector pNL4-3.Luc.R-E- [49] using a Lipofectamine3000 transfection reagent (Invitrogen). The cell culture medium was replaced after 6 h. After 24 h, the culture supernatant containing the HIV-pseudotyped virus with G and F proteins were collected. The supernatants were centrifuged at 3000 × g to remove cell debris, filtered through a 0.45 μm pore-size filter, and then stored at −80 °C.

### 2.9. Pseudovirus Neutralization Assay

To determine the neutralization ability of antibodies raised with the different G proteins, the sera were incubated for 30 min at 56 °C. The pseudovirus-containing supernatants were incubated with serially diluted sera at 37 °C for 1 h and added to 2 × 10^5^ pre-plated 293T cells in 96-well culture plates with two replicates per dilution. The cells were maintained in a high-glucose DMEM (Gibco) supplemented with 10% FBS (Gibco), penicillin (100 IU/mL), and streptomycin (100 μg/mL) at 37 °C with 5% CO_2_. After 48 h, the cells were lysed with a 20 μL cell lysis buffer (Promega, Madison, WI, USA). Next, 100 μL luciferase substrate (Promega) was added to the plates, and the relative luciferase activity was determined. The inhibition of the pseudovirus is presented as the percentage of inhibition in relative light units (RLUs). The IC50 was defined as the serum dilution at which the relative light units (RLUs) were reduced by 50% compared with the virus control wells (virus + cells). In the neutralization test using a monoclonal antibody, M102.4 was diluted three-fold starting from 10μg/mL with three replicates per concentration. The inhibition rates for each concentration were calculated and used to draw the curves.

### 2.10. Biolayer Interferometry Assay

Fc2HNV binding to the ephrinB2 receptor was measured using an Octet RED 96 system (Pall fortéBIO Corp, Menlo park, CA, USA). Data were acquired and analyzed using the kinetics mode of the Data Acquisition Software v9.0 (Pall fortéBIO Corp) or the Data Analysis Software v9.0 (Pall fortéBIO Corp). This method employed five steps: baseline, loading, baseline, association, and dissociation. Each step was done for 100 s, 180 s, 60 s, 300 s, and 600s separately. The recombinant mouse ephrin-B2 Fc chimera biotinylated protein (R&D systems) was diluted to 50 ng/μL with PBS and loaded into high-precision streptavidin (SAX) biosensors. G_NiV_, G_HeV_, and Fc2HNV were diluted to 500 nmol/mL with PBS. PBS-loaded biosensors were used as the reference sensors. Data of the reference sensors was subtracted from that of the experimental sensors. Then, the binding curves were derived. The curves were aligned by the baseline in step 3.

## 3. Results

### 3.1. Phylogenetic Analysis and Expression of G Proteins

**Figure 1:**
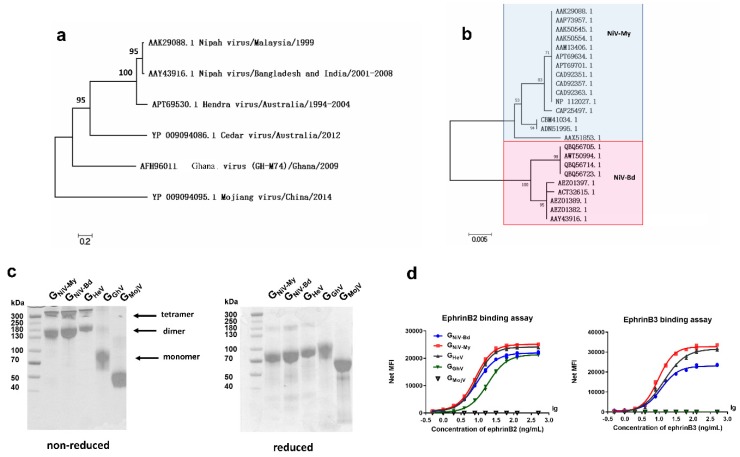
Phylogenetic analysis and expression of soluble G proteins. Molecular phylogenetic analysis of (**a**) henipaviruses-G glycoproteins (HNV-G) and (**b**) Nipah virus-glyco proteins (NiV-G). (**c**) G_NiV-My_ (G protein of Malaysia Nipah virus), G_NiV-Bd_ (G protein of Bangladesh Nipah virus), G_HeV_ (G protein of Hendra virus), G_GhV_ (G protein of Ghana virus), and G_MojV_ (G protein of Mojiang virus) were expressed in the Expi293 suspension cells and purified by affinity chromatography. Purified proteins were assessed on non-reduced and reduced SDS-PAGE gel. (**d**) G proteins are coupled to luminex magnetic microspheres, and the binding between G_NiV-My_, G_NiV-Bd_, G_HeV_, G_GhV_, and G_MojV_ and ephrinB2 or B3 receptors was then detected using the luminex xMAP multi-factor detection assay. The median fluorescence intensities (MFI) binding curves of the G proteins to receptors are shown. Each concentration contains two replicates, and each replicate is shown.

We first studied the phylogeny of the HNV-G proteins. To analyze the sequence similarity, we obtained 54 amino acid sequences of the HNV G proteins from GenBank, aligned them, and generated a G protein molecular phylogenetic tree using the maximum likelihood method (Figure 1a). The amino acid homology between the G proteins of HeV and NiV-My was 79%, whereas the G proteins of CedV, GhV, and MojV had 33%, 28%, and 20% amino acid homology with the NiV-My G protein. A total of seven HeV G protein sequences were aligned, and the amino acid homology was >99.5%. After aligning 24 sequences of the NiV-G protein, the Nipah viruses (NiVs) found in Malaysia and South Asia were found to belong to two different evolutionary branches: NiV-My and NiV-Bd, with an amino acid homology of 95% (Figure 1b).

We then expressed the HNV-G proteins of different evolutionary clades. G_NiV-My_, G_NiV-Bd_, G_HeV_, G_GhV_, and G_MojV_ were expressed in the Expi293 suspension cells and purified by affinity chromatography. Due to the presence of cysteine residues in the stalk region, G proteins may form dimers and tetramers on the surface of the viral envelope. When separated on a non-reduced SDS-PAGE gel, G_NiV-My_, G_NiV-Bd_, and G_HeV_ mostly formed dimers and tetramers, whereas G_GhV_ and G_MojV_ were primarily monomeric (Figure 1c). Due to the presence of multiple glycosylation sites, G proteins showed diffuse bands under SDS-PAGE.

We used a multiplex microsphere assay [50] to test the interactions between the receptors and G proteins. G_NiV-Bd_, G_NiV-My_, and G_HeV_ bound to ephrinB2 and ephrinB3 in a dose-dependent manner (Figure 1d). GhV presented a lower amino acid homology with the existing HNVs. G_GhV_-coupled microspheres bound to ephrinB2 in a dose-dependent manner and did not bind to ephrinB3 (Figure 1d). No fluorescence signal was detected with the addition of ephrinB2 or ephrinB3 to the G_MojV_-coupled microspheres, indicating that G_MojV_ does not bind to either ephrinB2 or ephrinB3 (Figure 1d).

Taken together, we studied the phylogeny of the HNV-G proteins and expressed five soluble G proteins belonging to different evolutionary clades. The soluble G proteins retain many native characteristics, including oligomerization and the ability to bind to ephrin receptors.

### 3.2. HNV-G Proteins Elicited Limited a Cross-Reactive Specific Antibody Response

**Figure 2:**
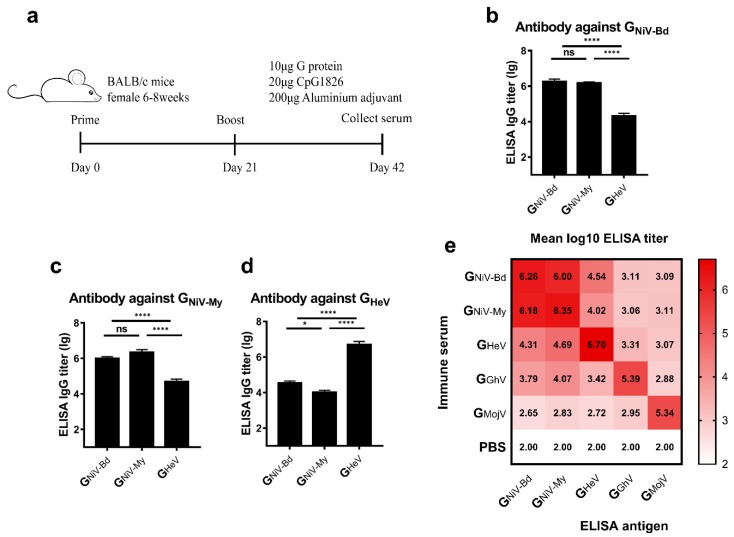
Cross-reactive specific antibody response induced by G proteins. (**a**) BALB/c mice (6–8 weeks, female, *N* = 10 per group) were immunized intramuscularly with 10 μg G_NiV-My_, G_NiV-Bd_, G_HeV_, G_GhV_, G_MojV_, or PBS at weeks 0 and 3. The adjuvants added were 200 μg of aluminum hydroxide and 20 μg of CpG1826. At 42 days after immunization, the mice were sacrificed, and the serum was collected. Specific antibodies against G proteins in the serum were detected by an enzyme-linked immunosorbent assay (ELISA). (**b**) Antibody titers against G_NiV-Bd_. (**c**) Antibody titers against G_NiV-My_. (**d**) Antibody titers against G_HeV_. The mean log10 ELISA titer ±SEM is shown. Student’s *t* test was performed for all comparisons, and a *P*-value < 0.05 was considered statistically significant; * 0.01< *P*-value < 0.05; **** *P*-value < 0.0001. (**e**) Matrix graph of the cross-reactive antibody responses elicited by the HNV-G proteins. The data are presented as the mean log10 ELISA titers. Same data is used in more than one panel.

To study the cross-reactive antibody response elicited by the G proteins in mice, we used the G proteins of HNVs to immunize mice with aluminum hydroxide and CpG1826 oligodeoxynucleotides (Figure 2a) and evaluated the cross-reactive antibody response of these G proteins by detecting specific antibodies.

We first compared the cross-reactive antibody reactions of the two clades of NiV. Serum from the G_NiV-My_ and G_NiV-Bd_ immunized groups had similar specific antibody titers against G_NiV-My_ and G_NiV-Bd_, respectively (Figure 2b,c).

We then analyzed the cross-reactive antibodies stimulated by G_HeV_ and G_NiV_. In the ELISA against G_NiV-Bd,_ the mean titer of the G_NiV-Bd_ group was approximately 89-fold higher than that of the G_HeV_ group. The titer of G_NiV-My_ group is also significantly higher than that of G_HeV_ group (Figure 2b). The G_HeV_ group elicited G_HeV_-specific antibodies with a mean log10 titer of 6.7, which was approximately 144-fold higher than that of the G_NiV-Bd_ group (Figure 2d), and also significantly higher than the G_NiV-My_ group (Figure 2d).

The mean log10 antibody titer of the G_GhV_-immunized serum against G_GhV_ was 5.39, and the cross-reactive antibody log10 titers against the other G proteins were lower (2.88–4.07) (Figure 2e). The mean log10 antibody titer of the G_MojV_-immunized serum against G_MojV_ was 5.34, whereas the log10 titers against other G proteins were between 2.66–2.95 (Figure 2e).

Taken together, the G proteins of NiV, HeV, GhV, and MojV elicited potent specific antibody responses and limited cross-reactive specific antibody responses.

### 3.3. HNV-G Proteins Elicited a Limited Cross-Reactive Neutralizing Antibody Response

#### 3.3.1. Receptor Competitive Inhibition Assay

To evaluate the cross-neutralizing antibody response stimulated by the G proteins, we used a luminex receptor competitive inhibition assay [50] to detect the neutralizing antibodies in the serum.

**Figure 3:**
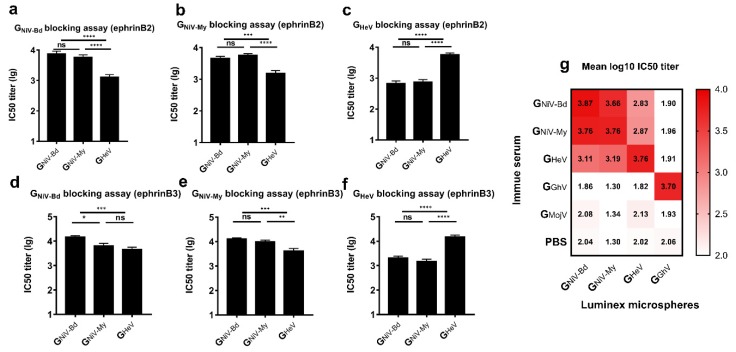
Cross-reactive neutralizing antibody response induced by G proteins. EphrinB2 competitive inhibition experiment against (**a**) G_NiV-Bd_, (**b**) G_NiV-My_, and (**c**) G_HeV_. EphrinB3 competitive inhibition experiment against (**d**) G_NiV-Bd_, (**e**) G_NiV-My_, and (**f**) G_HeV_. The data are presented as the mean log10 IC50 titer ±SEM. Student’s t test was performed for all comparisons, and a *P*-value < 0.05 was considered statistically significant; * 0.01< *P*-value < 0.05; ** 0.001< *P*-value < 0.01; *** 0.0001< *P*-value < 0.001; **** *P*-value < 0.0001. (**g**) Matrix graph of the EphrinB2 competitive inhibition experiment. The data are presented as the mean log10 IC50 titer. The same data is used in more than one panel.

In the ephrinB2 inhibition assay, G_NiV-My_ and G_NiV-Bd_ exhibited sufficient cross-protection, while no significant difference was observed in the IC50 titers between the two groups (Figure 3a,b). In the ephrinB3 inhibition assay against G_NiV-Bd_, the IC50 titer of the G_NiV-Bd_ group was higher than that of G_NiV-My_ (Figure 3d). In the ephrinB3 inhibition assay against G_NiV-My_, no significant difference was observed in the IC50 titers between the two groups (Figure 3e).

In the ephrinB2 inhibition assay against G_NiV-Bd_, the mean log10 IC50 titer of the G_NiV-Bd_ group was approximately 5.8-fold higher than that of the G_HeV_ group, with the mean log10 IC50 titer of the G_NiV-My_ group also being significantly higher than that of the G_HeV_ group (Figure 3a). In the ephrinB3 inhibition assay against G_NiV-Bd_, the mean log10 IC50 titer of the G_NiV-Bd_ group was 4.17, approximately 3.2-fold higher than that of the G_HeV_ group, and there was no significant difference between the G_NiV-My_ and G_HeV_ groups (Figure 3d).

In the ephrinB2 inhibition assay against G_HeV_, the mean log10 IC50 titer of the G_HeV_-immunized group was 3.76, approximately 8.5-fold higher than that of the G_NiV-Bd_ group and also significantly higher than that of the G_NiV-My_ group (Figure 3c). In the ephrinB3 inhibition assay against G_HeV_, the mean log10 IC50 titer of the serum from the G_HeV_-immunized group was 4.24, approximately 7.4-fold higher than that of the G_NiV-Bd_ group and also significantly higher than that of the G_NiV-My_ group (Figure 3f).

The serum of the G_GhV_-immunized group inhibited the binding of G_GhV_ to ephrinB2, with a mean log10 IC50 titer of 3.70 (Figure 3g). The serum of the G_GhV_ or G_MojV_ groups contained no cross-neutralizing antibodies against NiV/HeV. G_MojV_ also failed to elicit neutralizing antibodies against GhV.

#### 3.3.2. Pseudovirus Neutralizing Assay

**Figure 4:**
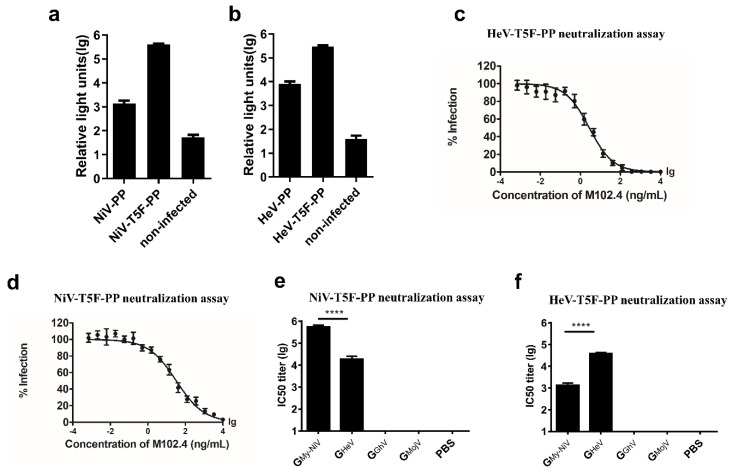
Packaging and verification of the NiV and HeV pseudoviruses. Codon-optimized, full-length G and F protein genes of NiV and HeV were inserted into the pcDNA3.1 vector to generate synthetic viral proteins. For the NiV-PP, the sequences of the G (Genebank protein ID: AAY43916.1) and F (AAY43915.1) proteins were used; for the HeV-PP, the sequences of the G (AEQ38071.1) and F (AEQ38070.1) proteins were used. By truncating the pcDNA3.1-NiV-F plasmid, 5 amino acids in the C-terminal intracellular region of the F protein were retained; the pseudovirus made by the truncated F plasmid was named NiV-T5F-PP. By truncating the pcDNA3.1-HeV-F plasmid, 5 amino acids in the C-terminal intracellular region of the F protein were retained; the pseudovirus made by the truncated F plasmid was named HeV-T5F-PP. (**a**) Effect of the truncation of the F proteins on the NiV pseudovirus titer. Each point represents three duplicates, and the error bar represents SEM. (**b**) Effect of the truncation of the F proteins on the HeV pseudovirus titer. (**c**) HeV pseudovirus-neutralizing experiment using M102.4. Each point represents three duplicates, and the error bar represents SEM. (**d**) NiV pseudovirus-neutralizing experiment using M102.4. (**e**) NiV pseudovirus-neutralizing experiment. (**f**) HeV pseudovirus-neutralizing experiment. The data are presented as the mean log10 IC50 ± SEM. Student’s t test was performed for all comparisons, and a *P*-value < 0.05 was considered statistically significant; **** *P*-value < 0.0001.

We further evaluated the neutralizing activity of the serum using a pseudovirus neutralizing assay [48,51]. Briefly, the genes encoding the full-length G and F proteins of NiV or HeV were inserted into the pcDNA3.1 vector and co-transfected into 293T cells together with the HIV backbone plasmid pNL4-3.Luc.R-E- (Figure 4a).

Studies have shown that truncation of the F protein can increase the titer of the NiV pseudovirus [48]. A truncated NiV-F protein, in which the intracellular region retains 5 amino acid residues, was constructed and transfected into 293T. The fluorescence signal of the pseudovirus with the truncated F protein was 276-fold higher than that of the wild type (Figure 4a). We then tested the same mutation on the HeV pseudovirus, and the fluorescence signal of the pseudovirus with the truncated F protein was 38-fold higher than that of the wild type (Figure 4b). The broad neutralizing antibody M102.4 can neutralize both NiV and HeV [44,45]. M102.4 inhibited the expression of the pseudoviral luciferase reporter gene in a dose-dependent manner (Figure 4c,d).

The above results indicate that we successfully packaged the pseudoviruses and that the expression of the pseudoviral luciferase can be inhibited by the neutralizing antibody M102.4; hence, we can use the pseudoviruses to evaluate the neutralizing antibodies in the serum.

In the NiV pseudovirus-neutralizing experiment, the mean log10 IC50 titer of the G_NiV_ group was 5.58, which was significantly higher than that of the G_HeV_ group (Figure 4e). The HeV pseudovirus-neutralizing experiment showed that the mean IC50 titer of the G_HeV_ group was 4.91, about 57.5 times higher than that of the G_NiV_ group (Figure 4f).

The serum of the G_GhV_ and G_MojV_ groups contained no cross-neutralizing antibodies against HeV/NiV according to the pseudovirus-neutralizing experiments (Figure 4e,f).

Taken together, the above quantitative studies on antibody responses show that different clades of NiV have sufficient cross-protection in mice, and that the G proteins of NiV and HeV elicited limited cross-neutralizing antibody responses. The G proteins of MojV and GhV were unable to elicit a cross-neutralizing antibody response against highly pathogenic HNVs, and there was no cross-protection between MojV and GhV.

### 3.4. Construction and Production of Bivalent Fc-Fusion Protein

**Figure 5:**
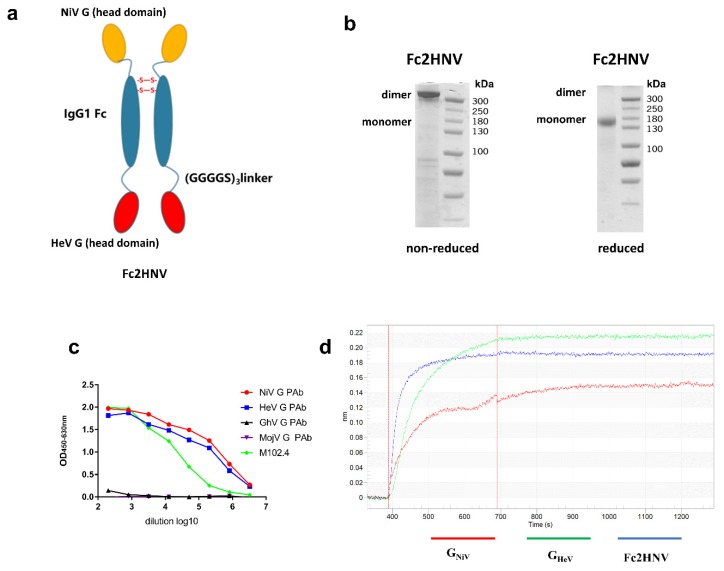
Construction and characterization of bivalent fc-fusion proteins. (**a**) Schematic diagram of the Fc-fusion protein Fc2HNV. The Fc2HNV contains the head domains of G_NiV_ and G_HeV_. The head domain of G_NiV_, the human IgG1-Fc domain, and the head domain of G_HeV_ were connected in sequence. These three domains were separated by two (Gly4Ser)3 flexible linkers. To obtain the secreted Fc-fusion protein, a tPA signal peptide was added at the N-terminus of the protein. (**b**) Fc2HNV assessed on a non-reduced and reduced SDS-PAGE gel. (**c**) Binding curves of Fc2HNV with M102.4 or pAbs against the G proteins of HNVs. The curves were determined by ELISA with the M102.4 diluted from 20 μg/mL. (**d**) Binding curves of Fc2HNV, G_NiV_, and G_HeV_ with the ephrinB2 receptor. The curves were determined by a biolayer interferometry assay. Data of the association and dissociation steps are presented, and all curves were aligned by a baseline step.

To elicit broad neutralizing antibodies against NiV and HeV, we generated an Fc-based recombinant protein. The fused protein, named Fc2HNV, contains the human IgG1- Fc domain with the head domains of G_HeV_ and G_NiV_ at its N-terminus and C-terminus, respectively. These three domains were separated by two (Gly4Ser)_3_ flexible linkers to ensure the correct protein folding and function (Figure 5a). In the SDS-PAGE experiment, Fc-2HNV showed a dimer and a monomer under non-reduced and reduced conditions, respectively (Figure 5b).

To assess the proper activity of the Fc-fusion protein, we tested its binding to the mAb, pAb, and ephrinB2 receptors. We used ELISA to characterize the binding between Fc2HNVand mAb, M102.4, or pAb against the HNV-G proteins (Figure 5c). Fc2HNV binds to M102.4, as well as pAb, against G_HeV_ and G_NiV_, but not pAb against G_GhV_ or G_MojV_. We used a biolayer interferometry (BLI) assay to characterize the binding between Fc2HNV and the ephrinB2 receptor. The BLI analysis showed that Fc2HNV binds to the ephrinB2 receptor (Figure 5d). This indicates that the G protein domains of the Fc-fusion proteins maintain antigenicity and conformation.

### 3.5. Bivalent Fc-Fusion Antigens Elicit a Broad Antibody Response against NiV and HeV in Mice

**Figure 6:**
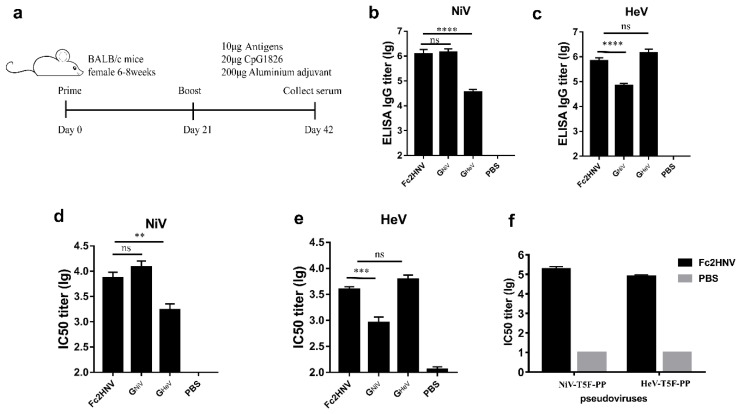
Specific antibodies and neutralizing antibody responses stimulated by Fc2HNV. (**a**) BALB/c mice (6–8 weeks, female, *N* = 6 per group) were immunized intramuscularly with 10 μg G_NiV_, G_HeV_ or Fc2HNV at week 0 and 3. The adjuvants added were 200 μg of aluminum hydroxide and 20 μg of CpG1826. A group of mice were injected with PBS as a control group. At 42 days after immunization, the mice were sacrificed and the serum was collected. Specific antibodies against G_NiV_ (**b**) and G_HeV_ (**c**) in the serum were tested by an enzyme-linked immunosorbent assay. Neutralizing antibody titers against NiV (**d**) or HeV (**e**) were detected by a multiplex microsphere ephrinB2 inhibition assay. (**f**) The NiV and HeV pseudovirus neutralization experiment. The mean log10 ELISA titers and mean log10 IC50 titers ±SEM are shown. Student’s *t* test was performed for all comparisons, and a *P*-value < 0.05 was considered statistically significant; ** 0.001< *P*-value < 0.01; *** 0.0001< *P*-value < 0.001; **** *P*-value < 0.0001.

We used Fc2HNV to immunize mice with aluminum hydroxide and CpG1826 oligodeoxynucleotides and evaluated the antibody response. For comparison, three groups of mice were immunized with the same dose of G_NiV_, G_HeV,_ or PBS, respectively (Figure 6a). As Fc2HNV has a larger molecular weight than G_NiV_ or G_HeV_, fewer moles of Fc2HNV were used. We found that Fc2HNV elicited potent specific and neutralizing antibodies against G_NiV_ and G_HeV_.

For the Fc2HNV group, the mean ELISA titer of the G_NiV_ specific antibodies was 6.08, which is significantly higher than that of the G_HeV_ group and similar to that of the G_NiV_ group (Figure 6b). The mean ELISA titer of the G_HeV_ specific antibodies was 5.84, which is significantly higher than that of the G_NiV_ group and similar to that of the G_HeV_ group (Figure 6c).

We evaluated the neutralizing antibodies using a luminex receptor competitive inhibition assay. The mean log10 IC50 titer against NiV elicited by Fc2HNV was 3.85, which is similar to that of the G_NiV_ group and significantly higher than that of the G_HeV_ group (Figure 6d). The mean log10 IC50 titer against the HeV elicited by Fc2HNV was 3.59, which is similar to that of the G_HeV_ group and significantly higher than that of the G_NiV_ group (Figure 6e). The group immunized with G_NiV_ or G_HeV_ elicited only a limited cross-reactive antibody response. In the pseudoviral neutralization experiments, Fc2HNV elicited neutralizing antibodies against NiV and HeV, and the IC50 titers of the neutralizing antibody were about 10^5^ (Figure 6f).

These data indicate that compared with G_NiV_ or G_HeV_, Fc2HNV can elicit a stronger broad neutralizing antibody response against both NiV and HeV.

### 3.6. Tetravalent Fc-Fusion Protein Triggers Broad Antibody Response against HNVs

**Figure 7:**
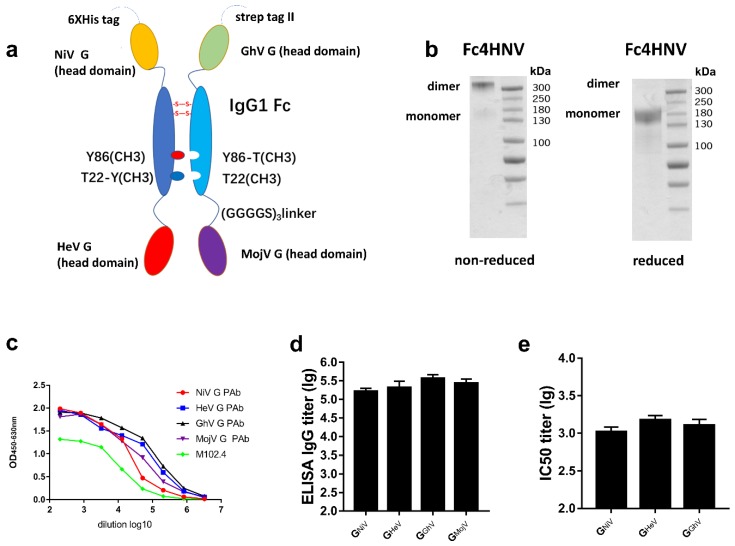
Specific antibodies and the neutralizing antibody response stimulated by Fc4HNV. (**a**) Schematic diagram of the Fc-fusion protein Fc4HNV. The Fc4HNV contains the head domains of G_NiV_, G_HeV_, G_GhV_, and G_MojV_. To obtain the secreted Fc-fusion protein, a tPA signal peptide was added at the N-terminus of the protein. To construct the pcDNA3.1-Fc4HNV-chainA plasmid, the head domain of G_NiV_, the human IgG1-Fc domain, and the head domain of G_HeV_ were connected in sequence and separated by two (Gly4Ser)3 flexible linkers. To construct the pcDNA3.1-Fc4HNV-chainB plasmid, the head domain of G_GhV_, the human IgG1-Fc domain, and the head domain of G_MojV_ were connected in sequence and separated by two (Gly4Ser)3 flexible linkers. The Fc domains of the two chains carry “knobs-into-holes” mutations. The pcDNA3.1-Fc4HNV-chainA and pcDNA3.1-Fc4HNV-chainB were co-transfected into Expi293 cells at a 1:1 ratio. (**b**) Fc4HNV assessed on a non-reduced and reduced SDS-PAGE gel. (**c**) Binding curves of Fc4HNV with M102.4 or pAbs against the G proteins of HNVs. The curves were determined by ELISA and the M102.4 diluted from 20 μg/mL. (**d**) BALB/c mice (6–8 weeks, female, N = 6 per group) were immunized intramuscularly with 10 μg Fc4HNV with aluminum hydroxide and CpG1826. Specific antibodies against HNV-G proteins in the sera tested by ELISA. (**e**) Neutralizing antibody titers against NiV, HeV, and GhV were detected by a multiplex microsphere ephrinB2 inhibition assay. The mean log10 ELISA titers and mean log10 IC50 titers ±SEM are shown.

To elicit a broad neutralizing antibody response against NiV, HeV, GhV, and MojV, we then designed a Fc-based recombinant protein named Fc4HNV, which consists of two parts (Figure 7a). One part is a chain that contains the human IgG1- Fc domain and the head domains of G_HeV_ and G_NiV_ at its N-terminus and C-terminus, respectively. The other part is a chain containing IgG1-Fc, the head domain of G_GhV_ and G_MojV_. The Fc domains of the two chains carry “knobs-into-holes” mutations, which can lead the two chains to form heterodimers [39].

In the non-reduced SDS-PAGE experiments, Fc4HNV was almost entirely present as a dimer (Figure 7b). We then used ELISA to characterize the binding between the Fc4HNV proteins and mAb, M102.4, or pAb against the G proteins. Fc4HNV binded to M102.4 and pAb against G_HeV_, G_NiV_, G_GhV,_ and G_MojV_ (Figure 7c).

After being immunized with Fc4HNV, the antibodies against the G proteins of NiV, HeV, GhV, and MojV were detected, with the mean log10 ELISA titers exceeding 10^5^ (Figure 7d). Neutralizing antibodies against NiV, HeV, and GhV were also detected, with the mean IC50 titers exceeding 10^3^ (Figure 7e). This indicates compatibility among the four antigens in the Fc-fusion protein. These results promote the use of the tetravalent Fc-fusion protein as a universal vaccine candidate, although further efficacy studies would be required.

## 4. Discussion

HNVs are emerging pathogens with high pathogenicity. So far, no vaccine has been approved for humans to prevent the diseases caused by HNVs. Considering the possibility of further potential large-scale outbreaks, broad-spectrum vaccines may be more effective than monovalent vaccines in preventing these diseases.

In order to rationally select the relevant antigens, we first investigated the cross-reactive antibody responses of the HNV G proteins. According to our results, although the time and geographical span of the NiV outbreaks are wide, the G proteins from the two viral genotypes had adequately protective effects. All human NiV outbreaks that occurred after 2000 were due to the Bangladesh strains, but there is also a recently described (2003) NiV isolate in Cambodia that belongs to the NiV Malaysia clade [6]. When developing the NiV vaccine using a G protein, the differences in strains may not affect the protective effect of the vaccine. This also indicates that the diseases caused by these two strains may not be distinguished by serological tests.

Furthermore, the amino acid homology between the HeV-G and NiV-G proteins is approximately 79%. The soluble G protein of HeV has been studied for more than a decade and is protective against NiV, as well as HeV, in ferrets and African green monkeys [24,52]. However, this vaccine failed to protect pigs from the NiV challenge, and the cross-neutralizing antibody levels against NiV did not reach protective levels [27].

In this study, the antibody titer against G_NiV_ for the group immunized with G_NiV_ was about 89-fold higher than that of the group immunized with G_HeV._ Additionally, the IC50 titers of the neutralizing antibody were significantly higher, which suggests that a single G protein may be insufficient to elicit a broad antibody response against NiV and HeV.

The amino acid homology between G_GhV_ and G_HeV_ is about 30%, and the amino acid homology between G_GhV_ and G_NiV-My_ is about 27%. EphrinB2 interacted with G_GhV_ and may be a cellular receptor for G_GhV_ [13]. Cross-neutralizing antibodies against NiV and HeV have been detected in two species of bats in Africa [53]. In *Pteropus* spp. in Ghana, the henipavirus antibody seroprevalence rate was as high as 40% [54]. As per the evidence, henipaviruses in bats have the risk of spill out. Cross-neutralizing antibodies against NiV and HeV have also been detected in residents of Cameroon [55,56]. A past study showed a panel of polyclonal and monoclonal antibodies against G_NiV_ that rarely bind to G_GhV_ [13]. Neither the Asiatic HNV-reactive nor the African HNV-reactive monoclonal antibodies exhibited cross-reactivity with G_MojV_ [28]. The co-expression of the MojV G and F proteins mediated the formation of syncytium in BSR-T7 cells; however, G protein cellular receptors have yet to be found [28].

Our results also indicate that there are no cross-neutralizing antibody responses between MojV and GhV and highly pathogenic HNVs (NiV/ HeV). Therefore, if GhV and MojV are pathogenic in humans, G_GhV_ or G_MojV_ could be used as a protective antigen, while the existing HNV vaccine candidates may not provide protection. Infection with GhV or MojV is unlikely to be the reason for the detection of NiV and HeV cross-neutralizing antibodies in African bats and human serum. Although no clinical cases of NiV or HeV infection have ever been reported in humans or animals in Africa, our study suggests that the species and distribution of the henipavirus in Africa requires further study.

Quantitative studies of the antibody responses elicited by the HNV-G proteins indicate that a single G protein may not be sufficient to elicit broad neutralizing antibodies against HNVs. In order to develop a universal vaccine, it may be necessary to combine the G proteins from different evolutionary clades. We demonstrated the feasibility of fusing different G proteins with IgG Fc to make multivalent vaccines.

In the research on HIV, respiratory syncytial virus, Epstein–Barr virus, and Ebola virus, fusion with Fc is helpful for improving the immune response stimulated by recombinant proteins [35,36,37,38]. The safety of Fc-fusion proteins has been proven by the use of Fc-based protein drugs and therapeutic monoclonal antibodies [33]. Due to the combination of Fc and the neonatal Fc receptor (FcRn), fusion with Fc can increase the plasma half-life of recombinant antigens [33]. Recombinant proteins can be purified using protein A/G affinity chromatography, thereby reducing the difficulty of obtaining antigens. Fusion with Fc may be beneficial for the stability of multivalent vaccines and may contribute to eliciting potent antibody responses.

In this study, we constructed a bivalent Fc-fusion vaccine containing the head domains of G_NiV_ and G_HeV_. Antigens were linked to the Fc backbone through flexible linkers, which reduced steric hindrance. The fused proteins were able to bind to the polyclonal antibodies and monoclonal antibody of G_NiV_ and G_HeV_. The fused protein bound to the ephrinB2 receptor, indicating that the original conformation of the fused G proteins was maintained in the fused protein. The Fc-fusion vaccine elicited a neutralizing antibody response against both NiV and HeV. Fc-fusion proteins are designed in a variety of forms, and most candidate vaccines based on Fc-fusion proteins fuse antigens to the N-terminus of the Fc domain [33,34,35,36,37]. Our bivalent fusion protein has shown that both the N-terminus and the C-terminus can carry the correct folded antigen and can stimulate a potent antibody response. The bivalent Fc-fusion protein forms a dimer, and although less moles of antigen are injected, the neutralizing antibody response elicited by the bivalent Fc-fusion protein is comparable to using G_NiV_ or G_HeV_ alone. Based on these results, we provide new options for broad-spectrum vaccines against these two fatal viruses.

The “knobs-into-holes” (KIHs) technology, which involves engineering CH3 domains to create either a “knob” or a “hole” in each heavy chain to promote heterodimerization, has been used in the construction of bispecific antibodies [39,57]. We want to use this technology to construct a universal vaccine against HNV.

Although no studies have confirmed the pathogenicity of GhV or MojV, once an outbreak occurs, no vaccine can be used to prevent it. Besides the bivalent Fc-fusion vaccine, we also constructed a heterodimeric Fc-fusion protein that fuses four G proteins of HNVs. The heterodimeric Fc-fusion protein backbone can carry four antigens. The fused antigen can be replaced as needed to make vaccines against multiple pathogens. Multiple antigens of a particular pathogen can also be combined to elicit a stronger immune response. The heterodimer tetravalent Fc-fusion protein was successfully expressed and formed a dimeric antibody-like molecule. This heterodimeric Fc-fusion vaccine can effectively elicit specific antibodies against the four G proteins and neutralizing antibodies against NiV, HeV, and GhV. Although the neutralizing antibodies against MojV cannot be evaluated yet, the high titers of specific antibodies suggest effective neutralization. There may also be some unrecognized pathogenic HNVs. MojV, GhV, NiV, and HeV belong to different evolutionary clades of HNVs. If a new HNV epidemic occurs, our vaccine candidate incorporating multiple G proteins is more likely to elicit broad neutralizing antibodies.

In animal experiments, we used the same dose of the Fc-fusion protein and monovalent vaccine. Due to the multiple antigen domains and Fc domains included in the Fc-fusion proteins, fewer of each antigen domain was actually injected in the immunization experiments. It is necessary to further evaluate whether higher injection doses can improve the effectiveness of multivalent vaccines. The neonatal Fc receptor (FcRn) participates in immune surveillance at mucosal barriers, such as in the intestine and lungs, through the bidirectional transport of IgG from the tissue interstitial space to the lumen, and vice versa [34]. A study shows that the FcRn/IgG transport pathway can be exploited to greatly enhance the efficacy of mucosally administered vaccines [58]. Further evaluation of whether the multivalent HNV vaccines can be administered through the mucosa may be valuable.

Taken together, our study clarified the cross-antibody reactions of various HNV-G proteins, providing useful data for advancing vaccine research. Concurrently, we expressed two forms of Fc-fusion HNV vaccine candidates. Our results reveal that both the bivalent and tetravalent HNV vaccines can elicit broadly neutralizing antibodies against HNVs, thereby representing a promising and broadly effective HNV vaccine candidate worthy of further development. To advance this vaccine candidate into the next development stage, its immunogenicity needs to be further characterized. For example, the effects of different doses and different vaccination strategies should be examined. Further evaluations of these vaccine candidates may be valuable to prepare us for possible HNV outbreaks in the future.
